# Duration of antimicrobial therapy in cardiovascular implantable electronic device associated systemic infections: a retrospective analysis

**DOI:** 10.1177/20499361261451368

**Published:** 2026-05-22

**Authors:** Emily Y. Xiao, Patrick T. Lynch, Sarwat Khalil, Derrick A. Draeger, Alexandra M. Lewis, Faiz M. Baqai, Mihail G. Chelu, M. Rizwan Sohail

**Affiliations:** Baylor College of Medicine Department of Internal Medicine 7200 Cambridge Street, Houston, TX 77030, USA; Department of Cardiology, Temple University, Philadelphia, PA, USA; Department of Medicine, Section of Infectious Diseases, Baylor College of Medicine, Houston, TX, USA; Department of Internal Medicine, Baylor College of Medicine, Houston, TX, USA; Department of Internal Medicine, Baylor College of Medicine, Houston, TX, USA; Department of Internal Medicine, Baylor College of Medicine, Houston, TX, USA; Department of Medicine, Section of Cardiology, Baylor College of Medicine, Houston, TX, USA; Cardiovascular Research Institute, Baylor College of Medicine, Houston, TX, USA; Texas Heart Institute at Baylor College of Medicine and Baylor St. Luke’s Medical Center, Houston, TX, USA; Department of Medicine, Section of Infectious Diseases, Baylor College of Medicine, Houston, TX, USA

**Keywords:** antibiotic duration, bacteremia, endocarditis, ICD, lead extraction, pacemaker

## Abstract

**Background:**

While the need for complete device removal is well-established for cardiovascular implantable electronic device (CIED) infections, there is insufficient evidence on the optimal duration of antimicrobial therapy.

**Objectives:**

To examine how antimicrobial duration affects clinical outcomes of patients presenting with CIED-associated bacteremia and lead endocarditis.

**Design:**

This is a retrospective cohort study conducted at a quaternary care hospital in Houston, TX, USA.

**Methods:**

Patients who underwent device removal for a primary indication of bacteremia or lead endocarditis over a 10-year period were stratified by prescribed duration of antibiotics as ⩽2 weeks or >2 weeks post-extraction. Measured outcomes included all-cause mortality at 90 days, recurrent bacteremia, infectious complications, and hospital length of stay.

**Results:**

Of 747 patients who underwent lead extraction at Baylor St. Luke’s Medical Center between June 2013 and December 2023, 79 cases met the inclusion criteria. Baseline characteristics were similar between cohorts. Mean duration of antibiotics prescription was 12.6 versus 38.6 days. There was no observed difference in survival (HR 0.693, 95% CI 0.085–5.652, *p* = 0.438), recurrent bacteremia (7% vs 6%, *p* = 0.952), infectious complications (27% vs 30%, *p* = 0.817), and hospital length of stay (mean 9.9 vs 13.3, *p* = 0.360) between the ⩽2 weeks and >2 weeks cohort, respectively. Five patients had recurrence, all of whom had bacteremia from *Staphylococcus aureus* or *Serratia* sp. and an underlying left ventricular assist device or valve replacement.

**Conclusion:**

Shorter duration of antibiotics after complete CIED removal had similar rates of mortality or recurrent bacteremia. Recurrence was associated with bacteremia from high-risk organisms with potential for secondary seeding of other cardiovascular prostheses. Larger prospective studies are needed to explore these findings.

## Plain language summary

A study of how long antibiotics could be used to treat infections of pacemakers and defibrillators

Why was the study done? Bacterial infections in the blood and infected pacemaker or defibrillator wires in the heart pose major health concerns in people who have them. If infected, these patients should have their devices removed and be treated with antibiotics to prevent further complications and even death. Most bacterial infections of the blood alone are treated with ⩽2 weeks of antibiotics, and most bacterial infections of heart valves require 4-6 weeks of antibiotics, but very little is known about how long to treat infections of pacemaker and defibrillator wires. This study is the first to explore how long antibiotics should be given for pacemaker and defibrillator wire infections.

What did the researchers do? The research team looked at all patients who had pacemaker or defibrillator wires removed due to a bacterial infection in the blood and/or infection of a pacemaker or defibrillator wire over a 10-year period at a hospital in Houston, TX. They grouped these patients by if they were prescribed ⩽2 weeks or >2 weeks of antibiotics and then compared rates of complications and death.

What did the researchers find? Of the 747 patients who had pacemaker or defibrillator wires removed over this period, 79 of them had a bacterial blood infection and/or infection of the wires. There was no difference in rates of death, length of hospitalization, or recurrent infection between people prescribed ⩽2 weeks versus >2 weeks of antibiotics. Interestingly, all 5 patients with recurrent infections had the same two types of bacteria (Staphylococcus aureus and Serratia) and prior heart surgeries.

What do the findings mean? This study provides some initial insight that shorter lengths of antibiotics may be appropriate for certain patients with pacemaker or defibrillator wire infections. However, patients with high-risk bacteria or certain prior heart surgeries may be at higher risk for recurrent infections. Larger studies are needed to explore these findings.

## Introduction

Infection is the most common indication for transvenous lead extraction of cardiovascular implantable electronic devices (CIED). Patients with CIED infections have higher long-term mortality compared to device recipients without infection, and those requiring complete hardware removal for source control of major CIED infections have a 5-fold higher risk of all-cause mortality.^1,2^ While there is strong evidence to support the role of complete system removal in the management of major CIED infection, evidence regarding the duration of antimicrobial therapy in CIED-associated systemic infections is lacking and is largely inferred from other studies related to the management of bloodstream infections, central-line associated bacteremia, and valvular endocarditis.^3^ Moreover, antibiotic prescribing practices are highly variable for these infections. In the 2024 American Heart Association Scientific Statement on CIED infections, expert consensus suggests that bloodstream infections should be treated with a longer duration of antibiotics, for at least 2 weeks, compared to the 10-day course suggested for CIED pocket infections without systemic involvement.^4^ For CIED-associated valvular infective endocarditis, 4–6 weeks of parenteral antimicrobial therapy is recommended.^4^ However, for patients with lead vegetations without valvular involvement, a shorter course of 2 weeks for non-*Staphylococcus aureus* (*S. aureus*) infection and 4 weeks for *S. aureus* after CIED removal is suggested.^3,5,6^ Longer courses of therapy for *S. aureus* bacteremia, even in otherwise uncomplicated cases, are typically recommended in recognition of this organism’s propensity for occult metastatic seeding and associated higher risk of relapse. As there are no randomized controlled trials evaluating the optimal duration of antibiotic therapy for various CIED infections, guidelines rely heavily on expert opinion and consensus. This exploratory, retrospective chart review aimed to analyze short-term mortality and recurrence of bacteremia among other clinical outcomes, for CIED-associated bacteremia and lead-related endocarditis patients who were prescribed varying durations of antibiotics.

## Methods

### Patient population and data acquisition

The patient cohort was generated for this retrospective chart review by manual review of all patients who underwent complete CIED system removal at Baylor St. Luke’s Medical Center in Houston, Texas, between June 2013 and December 2023. We included all patients who underwent CIED device extraction for a primary indication of treating a bloodstream infection with or without lead endocarditis. Patients who underwent extractions for noninfectious indications and pocket infections in the absence of bacteremia were excluded. Patients with valvular endocarditis were excluded, as there are existing evidence-based guidelines for the duration of treatment in valvular endocarditis.^3,6^ For the remaining patients included in the study, demographic, clinical, procedural, microbiologic and outcomes data were manually obtained from patient electronic medical records. This protocol was approved by the Baylor College of Medicine institutional review board. The reporting of this study conforms to the Strengthening the Reporting of Observational Studies in Epidemiology (STROBE) statement.^7^

### Definitions and outcomes

CIED systemic infection was defined as culture confirmed device-associated bacteremia with or without evidence of CIED lead-related endocarditis. To distinguish true bacteremia from blood culture contamination, we applied a definition of contamination previously described by Bekeris et al.^8^ A blood culture was considered to be contaminated if one or more of the following were identified in only one bottle of a series of blood culture specimens: coagulase-negative Staphylococcus species, Propionibacterium acnes, Micrococcus species, viridans-group streptococci, Corynebacterium species, or Bacillus species. A blood culture series was defined as one or more specimens collected serially within a 24-h period to detect an episode of bacteremia. Patients with contaminated blood culture specimens were not included as incident cases.

Duration of antibiotic therapy was stratified by less than or equal to 2 weeks (⩽2 weeks) or at least 2 weeks (>2 weeks) after the date of complete CIED removal based on published guidelines and practice patterns. Using an assigned treatment approach, we considered the duration of antibiotic therapy prescribed rather than the duration received to avoid biasing survival against patients who died prior to receiving the full duration of their antibiotic therapy. Measured outcomes included rates of recurrent bacteremia, infectious complications, post-operative disposition to intensive care unit (ICU), post-operative cardiac arrest, and all-cause mortality at 90 days post-extraction. Recurrent (relapse) bacteremia was defined as growth of the same species isolated from the prior CIED infection, detected on repeat blood cultures obtained between 7 and 90 days after lead extraction. Differentiation between true relapse and reinfection was not possible due to the absence of genotyping data. Infectious complications represented a composite of post-operative recurrent bacteremia, development of an abscess, septic emboli, and septic shock.

### Statistical analyses

Qualitative variables were represented as counts and percentages, while continuous variables were represented with mean and standard deviation (SD), except when otherwise specified. Univariate analysis was performed by *t*-test, chi-squared test, and logistic regression analysis. Multivariable analysis was performed by logistic regression analysis, in which variables with a *p*-value of <0.2 on univariate analysis were serially incorporated by stepwise forward selection into the multivariate analysis if they lowered the Akaike Information Criterion and Bayesian Information Criterion. Kaplan-Meier and Cox Regression analysis were also performed, with the index date defined as the date of complete CIED removal. No corrections for multiple comparisons were performed. Survival analysis was censored at the date of patient death or the date of the last known patient contact. Throughout, *p*-values of <0.05 were considered statistically significant. All analyses were performed using Stata (StataCorp LLC, College Station, TX, USA).

## Results

### Cohort characteristics

**Figure 1.**
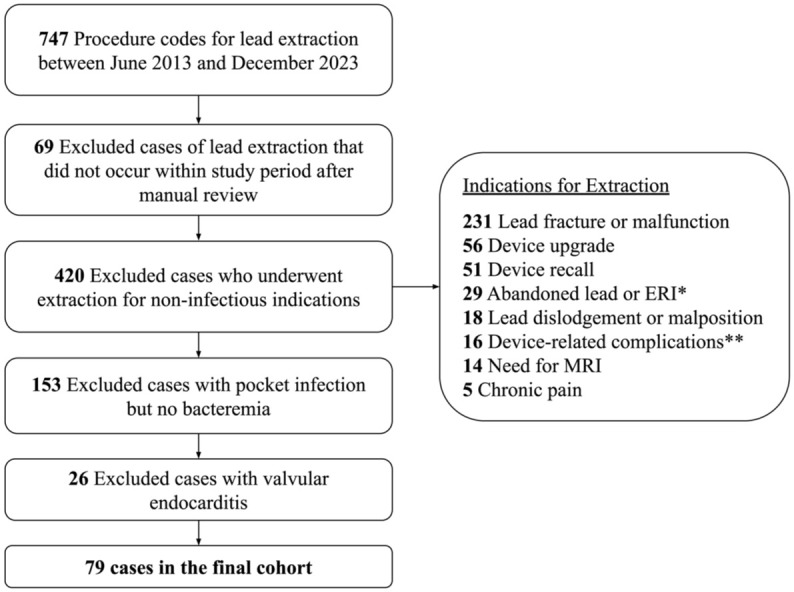
Flowchart of cohort creation. There were 678 cases of lead extractions performed at our institution over the 10-year study period. All 79 cases of complete device removal for cardiac implantable electronic device-associated bacteremia with or without lead vegetation were included in our analysis. *Elective Replacement Indicator (ERI) **10 venous occlusion or stenosis, 4 impingement or tethering of right ventricular lead to tricuspid valve, 1 pocket hematoma, 1 right ventricular lead erosion into the pericardium.

We reviewed 747 adult patients who underwent complete CIED removal during the study period, 668 of whom were excluded, resulting in a final cohort size of 79 patients who met our definition of CIED-associated systemic infection (Figure 1). Specifically, 69 cases were excluded because of device removal performed outside the specified time window, and 420 cases were excluded due to removal performed for noninfectious indications. The most common indications for CIED removal were infection (*n* = 258, 35%)—including pocket infections (*n* = 153, 21%), bacteremia (*n* = 32, 4%), valvular infective endocarditis (*n* = 26, 4%), and lead endocarditis (*n* = 47, 6%)—followed by lead fracture or malfunction (*n* = 231, 31%).

Baseline characteristics did not differ significantly between patients with antibiotic duration ⩽2 weeks and >2 weeks (Table 1). The majority of patients undergoing CIED removal for systemic infection had an implantable cardioverter defibrillator (*n* = 59, 75%) compared to a permanent pacemaker (*n* = 20, 25%). All patients underwent transesophageal echocardiogram (TEE), with lead vegetations visualized in 60% of patients (*n* = 47). Positron emission tomography/computerized tomography scanning (PET/CT) was performed in six patients (8%), and tagged white blood cell scan (tagged WBC) in two patients (3%). No patients underwent cardiac magnetic resonance imaging.

**Table 1.** Cohort characteristics.

Characteristics	Total (*n* = 79)	Antibiotic duration ⩽ 2 weeks (*n* = 15, 19%)	Antibiotic duration > 2 weeks (*n* = 64, 81%)	*p*-Value
Age	64.5 (13.4)	69.1 (13.2)	63.4 (13.3)	0.142
Sex				0.689
Female	23 (29%)	5 (33%)	18 (28%)	
Male	56 (71%)	10 (67%)	46 (72%)	
BMI	29.0 (7.5)	27.3 (7.9)	29.4 (7.4)	0.336
Device type				0.428
PPM	20 (25%)	10 (67%)	15 (23%)	
ICD	59 (75%)	5 (33%)	49 (77%)	
Lead age (years)	6.9 (4.1)	8.8 (5.1)	6.5 (3.8)	0.054
CAD	48 (61%)	7 (47%)	41 (64%)	0.214
Hypertension	70 (89%)	14 (93%)	56 (88%)	0.522
Hyperlipidemia	66 (84%)	13 (87%)	53 (83%)	0.717
Diabetes	42 (53%)	7 (47%)	35 (55%)	0.575
Tobacco	41 (52%)	6 (40%)	35 (55%)	0.305
CVA	0 (0%)	0 (0%)	0 (0%)	—
eGFR < 30	17 (22%)	5 (33%)	12 (19%)	0.216
ESRD	11 (14%)	4 (27%)	7 (11%)	0.113
OSA	23 (29%)	4 (27%)	19 (30%)	0.817
Cirrhosis	4 (5%)	1 (7%)	3 (5%)	0.753
CHD	2 (3%)	1 (7%)	1 (2%)	0.257
Heart failure	65 (82%)	10 (76%)	55 (85%)	0.079
LVEF	31 (13.5)	31.7 (13.7)	30.8 (13.6)	0.178
LVAD	15 (23%)	2 (20%)	13 (24%)	0.570
OHT	1 (2%)	1 (10%)	0 (0%)	—
Prosthetic valve	7 (9%)	0 (0%)	7 (11%)	0.180
HCM	3 (4%)	1 (7%)	2 (3%)	0.472
Afib	47 (59%)	8 (53%)	39 (61%)	0.587
VT	24 (30%)	5 (33%)	19 (30%)	0.782
Lead vegetation	47 (60%)	5 (33%)	43 (67%)	0.022
Pathogen				
*S. aureus*	36 (46%)	6 (40%)	30 (47%)	0.630
CoNS	14 (18%)	6 (40%)	8 (13%)	0.012
*Enterococci*	7 (9%)	2 (13%)	5 (8%)	0.498
Gram negative	11 (14%)	0 (0%)	11 (17%)	0.084
Other	11 (14%)	1 (7%)	10 (15%)	0.367
Days to extraction	7.3 (9.0)	6.4 (15.0)	7.5 (7.1%)	0.664
Preexisting hardware	36 (46%)	8 (53%)	28 (44%)	0.502
Antimicrobials				
Beta-lactams	50 (63%)	9 (60%)	41 (64%)	0.769
Vancomycin Daptomycin, Linezolid, or Clindamycin	32 (41%)	6 (40%)	26 (41%)	0.965
Other	15 (19%)	3 (20%)	12 (19%)	0.912

Baseline characteristics did not differ significantly between patients with antibiotic duration ⩽2 weeks and >2 weeks, other than more cases of lead vegetations in the cohort assigned to >2 weeks of therapy and a greater proportion of CoNS in the cohort assigned to ⩽2 weeks of therapy. Continuous variables are represented with means and standard deviations. Categorical variables are represented with count and percentage.

Afib, atrial fibrillation; BMI, body mass index; CAD, coronary artery disease; CHD, congenital heart disease; CoNS, coagulase-negative *staphylococci*; CVA, cerebrovascular accident; eGFR, estimated glomerular filtration rate; ESRD, end-stage renal disease; HCM, hypertrophic cardiomyopathy; LVAD, left ventricular assist device; LVEF, left ventricular ejection fraction; OHT, orthotopic heart transplantation; OSA, obstructive sleep apnea; VT, ventricular tachycardia.

Among patients with systemic CIED infections, 15 (19%) were prescribed ⩽2 weeks of antibiotic therapy for treatment of their infection compared to 64 (81%) who were prescribed >2 weeks of therapy. Mean duration of antibiotic therapy in those prescribed ⩽2 weeks of therapy was 12.6 days (1.82 weeks, SD 0.26 weeks), and median duration was 2 weeks (IQR 1.57–2 weeks). Mean duration of antibiotic therapy in those prescribed >2 weeks was 36.8 days (5.25 weeks, SD 1.32 weeks), and median duration was 6 weeks (IQR 4–6 weeks). Patients with lead-related endocarditis were less likely to be assigned ⩽2 weeks versus >2 weeks of antibiotics (33% vs 67%, *p* = 0.022). Patients with coagulase-negative *staphylococci* (CoNS) were more likely to be treated with ⩽2 weeks of antibiotics versus >2 weeks (40% vs 13%, *p* = 0.035).

### Procedural characteristics

Among all patients with systemic infections, the average number of days between hospital admission to CIED extraction was 7.31 days (SD 9.0) and did not differ significantly between the cohort who received ⩽2 weeks versus >2 weeks of antibiotics (6.4 vs 7.5, *p* = 0.664). The average lead age was 6.9 years (SD 4.1) between those who received ⩽2 weeks of antibiotics compared to those who received >2 weeks (8.8 vs 6.5, *p* = 0.054). The mean number of leads extracted was 2.4 leads per CIED, and a completely successful extraction of all leads was performed in 73 patients (92%). There were similar rates of successful device removal between those assigned ⩽2 versus >2 weeks of antibiotics (87% vs 94%, *p* = 0.351). Surgical extractions requiring sternotomy or thoracotomy occurred in four patients (5.1%), all of which were successful.

### Infectious characteristics

The most common organisms isolated in blood cultures were methicillin-resistant *S. aureus* (MRSA; *n* = 22, 28%), methicillin-sensitive *S. aureus* (MSSA; *n* = 14, 18%), and coagulase-negative *Staphylococci* (CoNS; *n* = 14, 18%). There was no difference in rates of *S. aureus* (40% vs 47%, *p* = 0.630) between cohorts. Of the 36 cases of *S. aureus*, 30 patients (83%) were prescribed >2 weeks of antibiotics, and 25 patients (69%) had lead vegetations versus 11 patients (31%) who had bacteremia only. There was a significantly greater proportion of CoNS in the group that was prescribed ⩽2 weeks of antibiotics compared to >2 weeks (40% vs 18%, *p* = 0.012). More detailed organism-specific analysis can be viewed in Supplemental Figures 1–3.

Multidrug-resistant organisms (MDRO) were identified in 14 patients (18%), and there was no association between the presence of MDRO and duration of antibiotic therapy prescribed (*p* = 0.575). Surgical cultures were obtained from either the CIED lead or device pocket in 70 patients (89%), of which 35 patients (44%) had both sites cultured. Surgical cultures yielded a pathogen in 38 patients (48%), and there were 13 patients (17%) who had different organisms isolated from blood cultures compared to surgical cultures.

The most common antibiotics prescribed in our patient cohort were cephalosporins (*n* = 45), vancomycin, daptomycin, linezolid, or clindamycin (*n* = 32), followed by tetracyclines (*n* = 10) and penicillins (*n* = 9). There were comparable rates of beta-lactam prescription (60% vs 64%, *p* = 0.769), nor in a composite of antibiotics (vancomycin, daptomycin, linezolid, clindamycin) that cover MRSA (40% vs 41%, *p* = 0.965) between patients who were assigned ⩽2 weeks versus >2 weeks of antibiotics. We did not analyze the transition from intravenous to oral step-down therapy, as institutional practice was for most patients to remain on intravenous antibiotics while hospitalized and transition to oral antibiotics at discharge, the timing of which may be influenced by extraneous factors that would bias the data.

Indwelling hardware or lines that could be other potential niduses of infection were present at the time of complete device removal in 36 patients (46%). This included intracardiac devices like left ventricular assist devices (LVAD) and septal occlusive devices (*n* = 14, 18%), valve replacements (*n* = 7, 9%), central venous catheters (*n* = 11, 14%), and orthopedic hardware (*n* = 11, 14%). The presence of indwelling hardware as a combined variable did not differ between patients who were prescribed ⩽2 weeks versus >2 weeks of antibiotics (53% vs 44%, *p* = 0.502). Likewise, there was no difference in duration of antibiotic therapy based on the presence of intracardiac devices (13% vs 19%, *p* = 0.621), prosthetic or mechanical valves (0% vs 11%, *p* = 0.180), or indwelling central lines (20% vs 13%, *p* = 0.450) when these categories were analyzed individually. More patients in the cohort prescribed ⩽2 weeks versus >2 weeks of antibiotics had orthopedic hardware (20% vs 9%, *p* = 0.016).

### Patient outcomes

**Figure 2.**
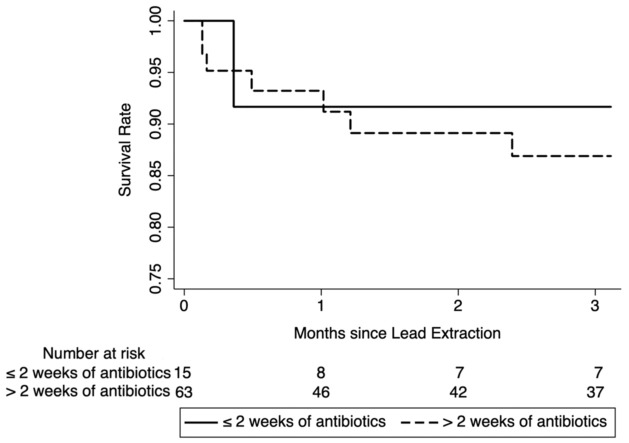
Survival stratified by duration of antibiotic therapy. Kaplan-Meier survival analysis showed no significant difference in mortality between patients who were prescribed less than 2 weeks versus at least 2 weeks of antibiotic therapy for cardiac implantable electronic device-associated bacteremia with or without vegetation (HR 0.693, 95% CI 0.085–5.652, *p* = 0.438). Patients in the cohort with shorter antibiotic duration had lower mortality, which may be attributable to clinicians prescribing shorter courses of therapy to patients who appeared to be more stable.

Overall survival of patients at 90 days following CIED hardware removal was 89% (Figure 2). Of the nine patients who died in this period, there was no observed difference in mortality between those who were prescribed ⩽2 weeks of antibiotics compared to those who were prescribed >2 weeks of antibiotics (7% vs 13%, *p* = 0.522). Univariate Kaplan-Meier survival analysis did not demonstrate a significant difference in survival at 90 days post-extraction (HR 0.693, 95% CI 0.085–5.652, *p* = 0.438), though patients who were prescribed the shorter duration of therapy had higher overall survival, which may represent prescriber bias. When survival analysis was repeated using the duration of antibiotic therapy actually received rather than prescribed, and after excluding those who died before the conclusion of therapy, there was similar mortality at 90 days post-device removal (*p* = 0.445).

Among the 36 patients with MSSA or MRSA bacteremia, mortality between treatment groups was comparable (17% vs 17%, *p* = 1.000) at 90 days as well. Only 1 out of 14 patients with CoNS bacteremia died within a year of extraction, and that patient was prescribed > 2 weeks of antibiotics. Only 2 out of 29 patients with non-staphylococcal bacteremia died within 90 days of complete device removal, and both were prescribed >2 weeks of antibiotics.

Univariate analysis revealed that the presence of lead vegetations, lower BMI, CAD, cirrhosis, history of ventricular tachycardia (VT), and history of atrial fibrillation were independent predictors of increased mortality (Table 2). On multivariate analysis, lower BMI (HR 0.83, CI 0.70–0.99, *p* = 0.037) and history of VT (HR 7.19, CI 1.09–47.81, *p* = 0.041) were the only variables that predicted higher mortality at 90 days. When the duration of antibiotic therapy was incorporated post-hoc into the multivariate analysis for exploratory purposes, it was not a significant predictor for 90-day survival (HR 0.41, CI 0.46–3.62, *p* = 0.420). However, with only nine deaths, multivariable Cox regression is statistically fragile, and the inclusion of multiple covariates risks model overfitting.

**Table 2.** Univariate and multivariate Cox regression predicting overall survival at 90 days.

Characteristics	HR, univariate (CI)	*p*-Value	HR, multivariate (CI)	*p*-Value
Antibiotics ⩽ 2 weeks	0.70 (0.09–5.65)	0.732	—	—
*S. aureus* bacteremia	2.01 (0.48–8.41)	0.339	—	—
Lead vegetation	4.55 (0.56–37.00)	0.156	2.06 (0.20–21.70)	0.548
Days to extraction	1.03 (0.97–1.11)	0.262	—	—
Incomplete extraction	1.57 (0.19–12.77)	0.674	—	—
Number of leads	0.91 (0.23–1.91)	0.797	—	—
Presence of hardware	1.61 (0.29–4.65)	0.832	—	—
Laser extraction	0.84 (0.10–6.81)	0.868	—	—
Age	1.02 (0.97–1.08)	0.444	—	—
Female sex	0.82 (0.17–4.06)	0.807	—	—
BMI	0.88 (0.77–1.00)	0.047	0.83 (0.70–0.99)	0.037
CAD	4.97 (0.61- 40.38)	0.134	3.82 (0.43–33.85)	0.228
Hypertension	—	—	—	—
Hyperlipidemia	1.33 (0.16–10.83)	0.789	—	—
Diabetes	2.54 (0.51–12.93)	0.252	—	—
ESRD	0.98 (0.12–7.99)	0.986	—	—
OSA	0.65 (0.13–3.22)	0.594	—	—
Decompensated cirrhosis	6.72 (1.35–33.40)	0.020	2.49 (0.45–13.77)	0.297
CHD	—	—	—	—
Heart failure	—	—	—	—
Advanced heart failure	0.77 (0.20–3.03)	0.708	—	—
Valve replacement	1.44 (0.18–11.73)	0.732	—	—
HCM	—	—	—	—
VT	5.50 (1.11–27.33)	0.037	7.19 (1.09–47.81)	0.041
Atrial fibrillation	5.92 (0.73–48.27)	0.097	5.45 (0.59–50.59)	0.136

Presence of lead vegetation, low BMI, CAD, decompensated cirrhosis, atrial fibrillation, and ventricular tachycardia were independent predictors of mortality by univariate analysis. Multivariate analysis found that only low BMI and ventricular tachycardia were associated with higher mortality.

BMI, body mass index; CAD, coronary artery disease; CHD, congenital heart disease; ESRD, end-stage renal disease; HCM, hypertrophic cardiomyopathy; OSA, obstructive sleep apnea; VT, ventricular tachycardia.

**Figure 3.**
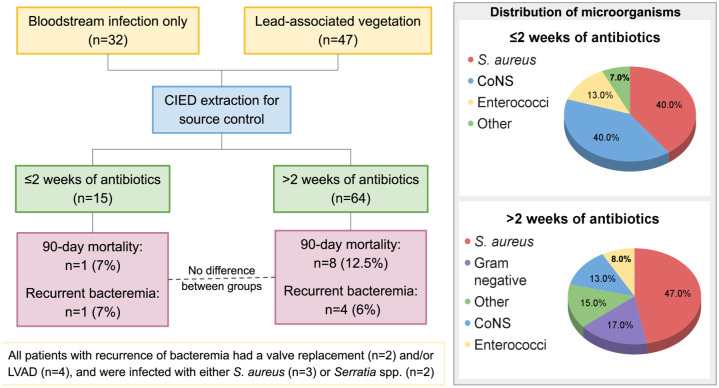
Management of cardiac implantable electronic device-associated systemic infections. The most common organism isolated in blood cultures from both cohorts was *S. aureus*. All five patients with recurrent bacteremia had an underlying cardiac prosthesis or were infected with *S aureus* or a *Serratia* species. There was no significant difference in mortality or recurrent bacteremia between those assigned to less than 2 weeks versus at least 2 weeks of antibiotic therapy. CIED, Cardiac implantable electronic device; CoNS, coagulase-negative staphylococci; LVAD, left ventricular assist device.

All five patients with recurrent bacteremia after complete CIED removal had prior valve replacements or LVADs, and all had either *S. aureus* or *S. marcescens* bloodstream infections (Table 3). Patients who were prescribed ⩽2 weeks of antibiotic therapy did not significantly differ in the rate of recurrent bacteremia when compared to those prescribed >2 weeks of therapy (7% vs 6%, *p* = 0.952) nor in the rate of infectious complications (27% vs 30%, *p* = 0.817; Figure 3). There were no significant differences in hospital length of stay by mean (9.9 vs 13.3, *p* = 0.360) nor median (9, IQR 6–14 vs 9 IQR 5.5–15), post-operative ICU disposition (0% vs 17%, *p* = 0.112), or rates of cardiac arrest (7% vs 5%, *p* = 0.577) between patients who were prescribed ⩽2 weeks of antibiotics versus >2 weeks of antibiotics, respectively.

**Table 3.** Descriptive analysis of patients with recurrent bacteremia after complete CIED removal.

Age, sex	Blood culture isolate	Vegetation vs bacteremia only	Weeks of antibiotics prescribed	Days between initial and recurrent blood culture	Other foci of infection
67 years, female	*Serratia marcescens*	Right atrial lead vegetation	Six followed by chronic suppression	68	mitral valve replacement, LVAD
74 years, male	MRSA	Right ventricular lead vegetation	1.3	39	LVAD
59 years, male	MRSA	Bacteremia only	Six followed by chronic suppression	42	LVAD, manubrium septic arthritis at time of extraction
61 years, female	*Serratia marcescens*	Bacteremia only	Eight followed by chronic suppression	54	LVAD, possible brain abscess post-device removal
43 years, female	MSSA	Bacteremia only	Six followed by chronic suppression	41	mitral valve replacement, possible infected central venous catheter at time of extraction

All five patients who had relapse or recurrence of bacteremia had either MRSA or *Serratia* and either an LVAD or valve replacement. These organisms are strongly associated with seeding of cardiovascular prosthetics.

LVAD, left ventricular assist device; MRSA, methicillin-resistant *Staphylococcus aureus*; MSSA, methicillin-sensitive *Staphylococcus aureus.*

## Discussion

### Clinical significance

While there is a growing body of published literature providing evidence-based guidance on management of various facets of CIED infections,^3,6,9^ data regarding optimal duration of antibiotic therapy in patients presenting with systemic CIED infection, with or without lead-related endocarditis, are lacking. Defining the optimal duration of antibiotic therapy for CIED infection is critical. Unnecessarily prolonged courses may be associated with drug toxicity, emergence of antimicrobial resistance, and line-related complications, whereas an inadequate duration of therapy may increase the risk of relapse and mortality. Our study represents the first attempt to examine how the duration of antimicrobial therapy affects outcomes for patients with CIED lead endocarditis after total CIED hardware extraction.

### Patient outcomes

Our study found that in cases of CIED-associated bacteremia and lead endocarditis, patients assigned to ⩽2 weeks versus >2 weeks of therapy after complete CIED removal had no observed difference in outcomes, including cases of MSSA and MRSA infections, though the small cohort size precludes our ability to draw conclusions about the safety or efficacy of abbreviated antibiotic courses in patients with *S. aureus* infections. This is consistent with our understanding that, unlike valvular endocarditis, complete hardware removal provides source control for lead-associated “endocarditis” and therefore these patients can potentially be managed with a shorter course of antibiotics after complete device removal rather than the 4–6 weeks of therapy recommended for valvular endocarditis.

There were no significant disparities in patient characteristics and procedural characteristics between the study cohorts. There were, however, two notable differences in the infectious characteristics between the groups. First, there was an unsurprising finding that a greater proportion of patients prescribed >2 weeks of antibiotics had CIED-associated lead vegetations, which, when labeled as lead-related “endocarditis”, may influence clinician prescribing behavior due to the terminology. Second, there was a greater proportion of CoNS in the cohort prescribed ⩽2 weeks of antibiotics, though all these cases were treated as true CIED-associated bacteremia and none were believed to be contaminants by the consulting infectious disease specialist based on the number of positive cultures and clinical assessment.

Interestingly, patients assigned to ⩽2 weeks of antibiotic therapy had overall lower mortality, though this difference was not statistically significant. This phenomenon may be explained in part by the prescribing provider’s bias to assign shorter durations of antibiotics to patients who appear more clinically stable and to assign longer courses of antibiotics to patients who appear to be sicker. Regardless, the absence of decreased survival among patients with shorter courses of antibiotics offers compelling preliminary evidence that abbreviated antibiotic courses may be appropriate in certain patients who are not at risk for secondary infectious syndromes from seeding of other preexisting hardware.

### Recurrent bacteremia

All five cases of recurrent bacteremia occurred exclusively in patients with intracardiac hardware, either a valve replacement or LVAD, and all involved either *S. aureus* or *Serratia* species. These findings are consistent with prior literature indicating these organisms are associated with a higher risk of seeding cardiovascular devices.^10^ This suggests that recurrence was likely driven by secondary seeding rather than insufficient antibiotic duration for CIED infection. An individualized approach to the evaluation and management of CIED infection—particularly regarding the need for additional imaging, duration of antibiotic therapy, and consideration for chronic suppressive treatment—is therefore warranted.

**Figure 4.**
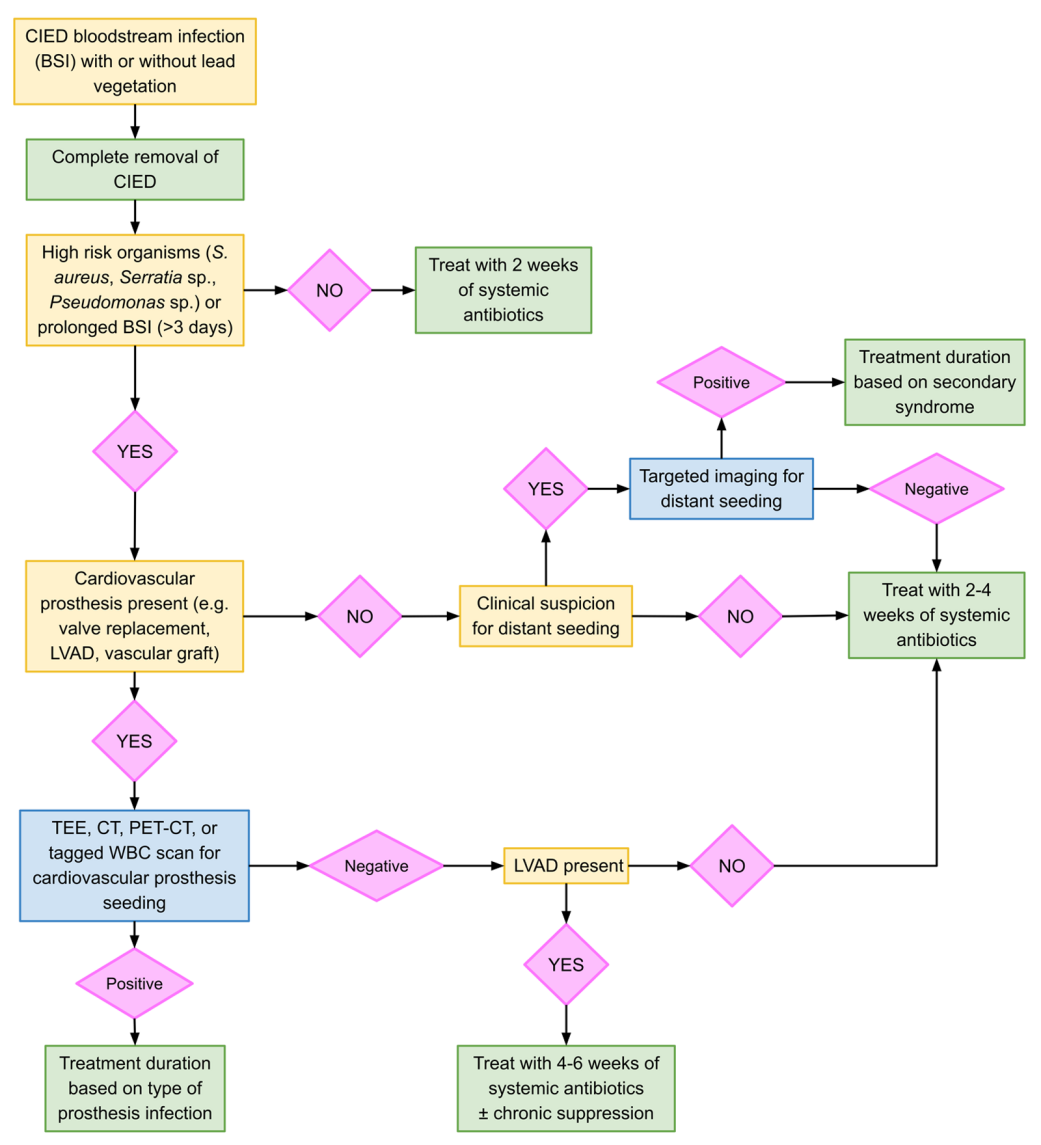
Proposed treatment algorithm based on expert opinion for CIED-associated bloodstream infections and lead-related endocarditis. Our proposed treatment algorithm suggests that patients with CIED infections not caused by high-risk organisms, and undergoing complete device removal, may be managed with 2 weeks of antibiotics. Patients with high-risk organisms should be evaluated for the presence of infection in other cardiovascular prostheses or distant seeding, and if identified, treatment duration should be extended and tailored to the specific infectious syndrome. CIED, cardiovascular implantable electronic device.

Based on our study data and clinical experience, we propose a management algorithm that combines our study findings (detection of high-risk organisms on blood cultures and presence of cardiovascular prostheses) with American Heart Association guidelines^3^ to determine the duration of antibiotic therapy and need for chronic suppression (Figure 4). However, larger studies are needed before this approach can be adopted into routine clinical practice.

### Study limitations

The prescribing bias, small sample size, and retrospective design limit causal inference based on this study alone. Sample size calculation and power analysis were not performed, creating uncertainty for the number of study subjects needed to reliably detect a statistically significant effect. The absence of severity-of-illness markers and propensity-score matching in a small cohort size precludes us from drawing any conclusions of equivalence or non-inferiority of shorter durations of therapy. Survival analysis utilized the duration of antibiotic therapy prescribed, and some patients were censored prior to the conclusion of their therapy. Our analysis did not reveal a statistically significant difference in mortality between antibiotic therapy for ⩽2 weeks compared to >2 weeks, but the survival curves do appear to separate over time and may possibly have yielded a statistically significant result with a larger cohort.

Moreover, nearly half of all patients had other potential niduses for infection at the time of CIED extraction, including various types of hardware, intracardiac devices, and indwelling catheters. While source control was thought to be achieved at the time of CIED extraction, there is a possibility that some patients may have had unknown seeding of other distant sites, requiring a longer duration of antibiotics or source control. PET/CT and tagged WBC scans were rarely performed at our institution, even in patients with pre-existing hardware, which raises concern for undetected occult secondary seeding that would have otherwise influenced decisions on antibiotic duration.

Our analysis was further limited by the binary nature of the study cohorts. Stratification of antibiotic duration (e.g. 3 weeks vs 4 weeks of therapy) was not performed, given the small sample size, which may obscure dose-response relationships. The timing of oral step-down therapy was not analyzed as an independent variable, as patients typically remained on intravenous antibiotics for the duration of the hospital stay and only transitioned to oral antibiotics at discharge, guided by the Infectious Disease service on a case-by-case basis, which affects the generalizability of the study findings due to variable institutional practices. Lastly, over the 10-year study period, antibiotic prescribing practices, lead extraction techniques, and guideline recommendations evolved. However, given the cohort size, we were unable to adjust for year of extraction or perform sensitivity analyses by treatment era.

### Future directions

Despite the afore-stated limitations, we believe that our data provide valuable initial insights into the management of CIED-associated bacteremia and lead vegetations without valvular involvement. Current guidelines regarding the duration of antimicrobial therapy in this population are largely founded on expert opinion rather than evidence. We believe that our preliminary data provide a compelling argument that an individualized approach based on identifying risk factors for recurrent infection or secondary seeding of other hardware is warranted. While our investigation demonstrated no association between patient outcomes and shorter durations of antibiotic therapy, the study was limited by cohort size, and therefore, larger, multicenter, prospective studies are needed to validate these observed associations.

## Conclusion

Among 79 patients with CIED-associated bacteremia or lead endocarditis who underwent complete device removal, there were similar rates of mortality, recurrence of bacteremia, and hospital length of stay between patients prescribed ⩽2 weeks compared to >2 weeks of antimicrobial therapy. Recurrent bacteremia occurred exclusively in patients with additional intracardiac hardware and high-risk organisms for prosthesis seeding, suggesting that recurrence was primarily due to secondary seeding rather than insufficient antibiotic duration for CIED infection. This data suggests that after source control is achieved, 2 weeks of antibiotic therapy may be adequate in select patients with CIED infections without other pre-existing hardware. While these study findings alone do not demonstrate equivalency or efficacy of shorter durations of antimicrobial therapy, they provide novel hypothesis-generating insights for future multicenter investigations.

## Supplemental Material

sj-docx-1-tai-10.1177_20499361261451368 – Supplemental material for Duration of antimicrobial therapy in cardiovascular implantable electronic device associated systemic infections: a retrospective analysis Supplemental material, sj-docx-1-tai-10.1177_20499361261451368 for Duration of antimicrobial therapy in cardiovascular implantable electronic device associated systemic infections: a retrospective analysis by Emily Y. Xiao, Patrick T. Lynch, Sarwat Khalil, Derrick A. Draeger, Alexandra M. Lewis, Faiz M. Baqai, Mihail G. Chelu and M. Rizwan Sohail in Therapeutic Advances in Infectious Disease

sj-docx-2-tai-10.1177_20499361261451368 – Supplemental material for Duration of antimicrobial therapy in cardiovascular implantable electronic device associated systemic infections: a retrospective analysis Supplemental material, sj-docx-2-tai-10.1177_20499361261451368 for Duration of antimicrobial therapy in cardiovascular implantable electronic device associated systemic infections: a retrospective analysis by Emily Y. Xiao, Patrick T. Lynch, Sarwat Khalil, Derrick A. Draeger, Alexandra M. Lewis, Faiz M. Baqai, Mihail G. Chelu and M. Rizwan Sohail in Therapeutic Advances in Infectious Disease

## Declarations

**Ethics approval and consent to participate:** This study protocol (H-52538) was approved by the Baylor College of Medicine institutional review board. The need for informed consent was waived by the institutional review boards as the study was retrospective, no patients were contacted throughout the duration of the study, and risks to patients were minimal.

**Consent for publication:** Not applicable.
