# Supplementary material for: Duration of antimicrobial therapy in cardiovascular implantable electronic device associated systemic infections: a retrospective analysis
Source: Ther Adv Infect Dis. 2026 May 22;13:20499361261451368. doi: 10.1177/20499361261451368 (PMC13198637; doi:10.1177/20499361261451368)
Supplement: sj-docx-2-tai-10.1177_20499361261451368 – Supplemental material for Duration of antimicrobial therapy in cardiovascular implantable electronic device associated systemic infections: a retrospective analysis [file sj-docx-2-tai-10.1177_20499361261451368.docx]

**Supplementary Material**

**Supplemental Figure 1: Mortality and Recurrence of Bacteremia in Patients with *Staphylococcus aureus* (*S. aureus)***


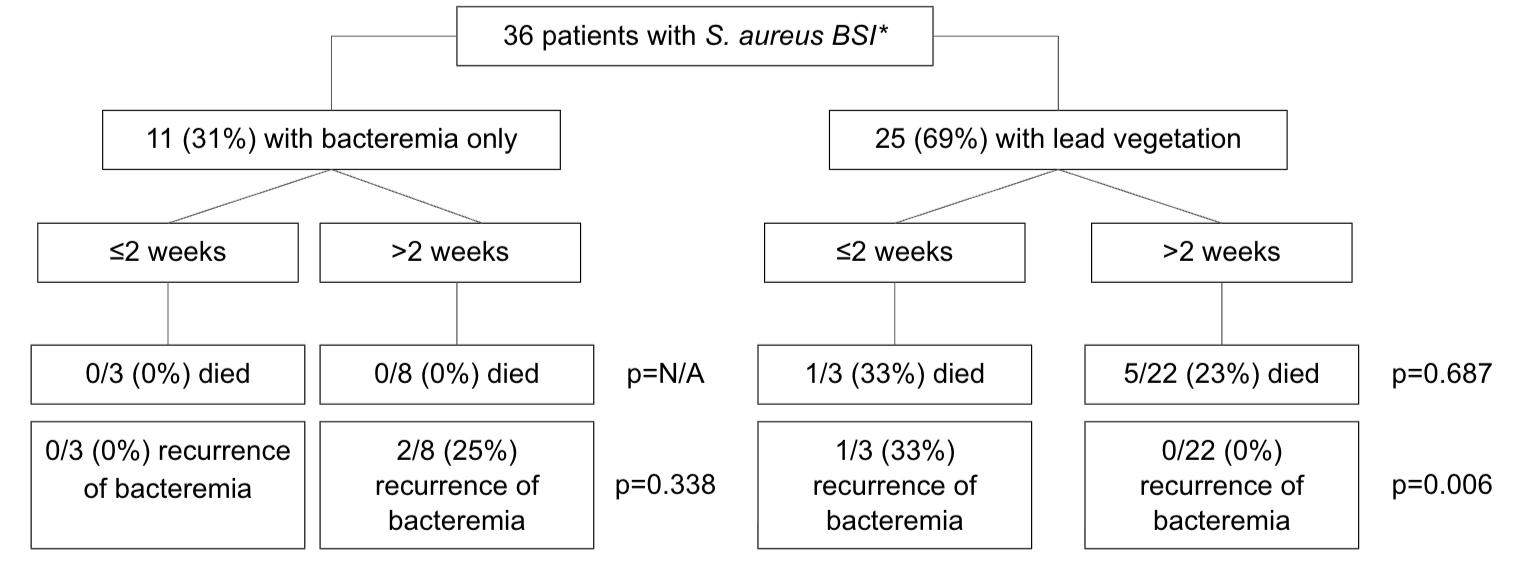
Mortality rate and recurrence of bacteremia performed by chi-squared test. Only accounting for deaths and recurrent bacteremia within 90 days post-extraction. Of the 36 patients with *S. aureus* bacteremia, there was no significant difference in mortality between patients assigned to ≤ 2 vs > 2 weeks of antibiotic therapy, but there was a higher recurrence of bacteremia in those with *S. aureus* associated lead vegetations in the ≤ 2 weeks cohort
*BSI = bloodstream infection

**Supplemental Figure 2: Mortality and Recurrence of Bacteremia in Patients with Non*-*** ***Staphylococcus aureus (S. aureus)* Gram Positive Cocci (CoNS, *Enterococcus s*p., *Streptococcus s*p.)**


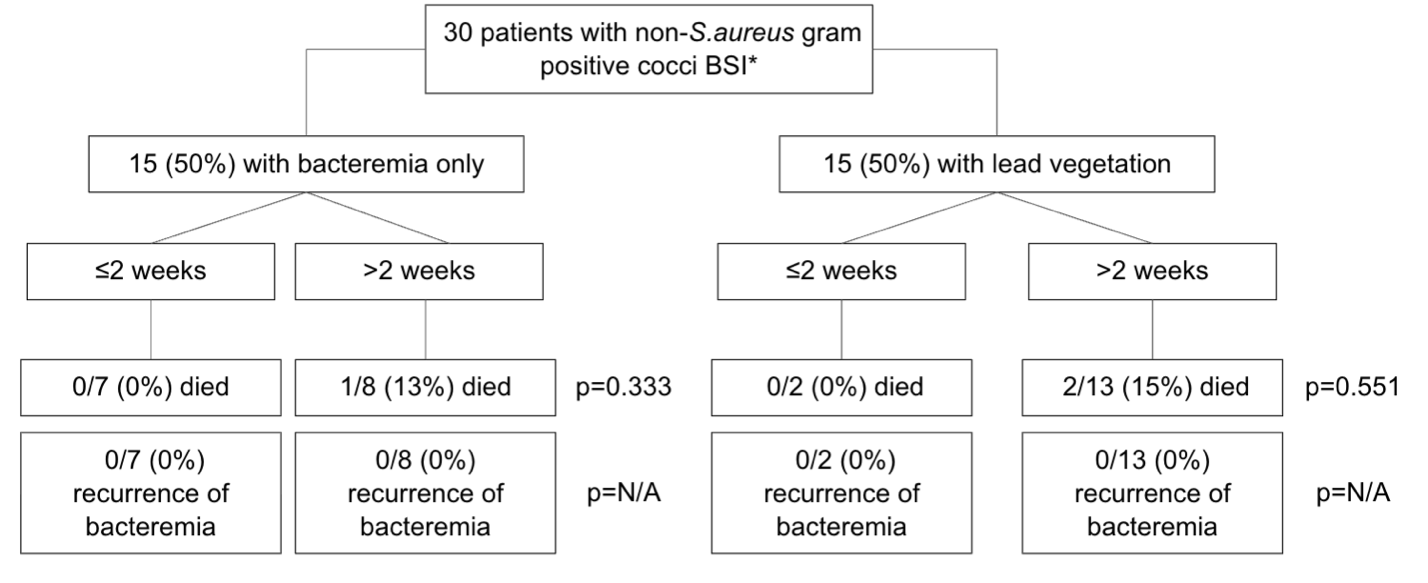
Mortality rate and recurrence of bacteremia performed by chi-squared test. Only accounting for deaths and recurrent bacteremia within 90 days post-extraction.  Of the 30 patients with gram positive cocci bacteremia, there was no significant difference in mortality between those assigned to ≤ 2 weeks vs. > 2 weeks of antibiotic therapy, and there were no cases of recurrence of bacteremia
*BSI = bloodstream infection

**Supplemental Figure 3: Mortality and Recurrence of Bacteremia in Patients with Gram-negative Organisms**


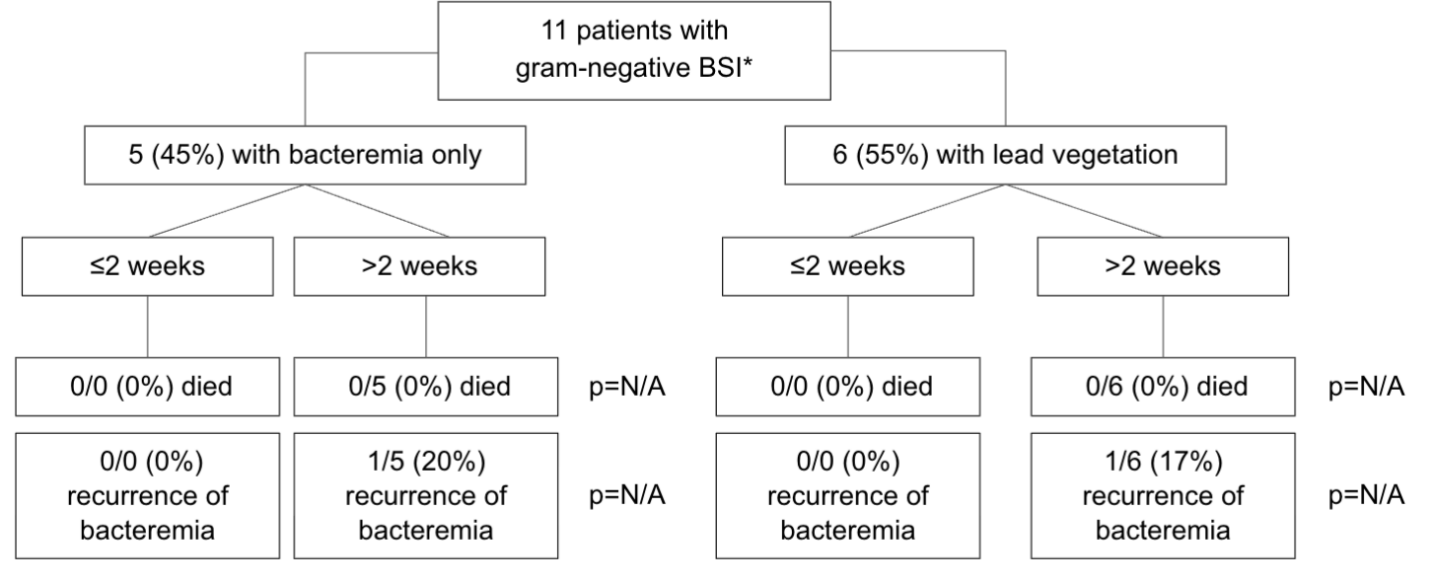
Mortality rate and recurrence of bacteremia performed by chi-squared test. Only accounting for deaths and recurrent bacteremia within 90 days post-extraction.  Of the 11 patients with gram-negative organism bacteremia, only 2 patients died and none had recurrence of bacteremia
*BSI = bloodstream infection
